# Supplementary figures and images for: Selective whole-genome amplification reveals population genetics of Leishmania braziliensis directly from patient skin biopsies
Source: PLoS Pathog. 2023 Mar 20;19(3):e1011230. doi: 10.1371/journal.ppat.1011230 (PMC10063166; doi:10.1371/journal.ppat.1011230)

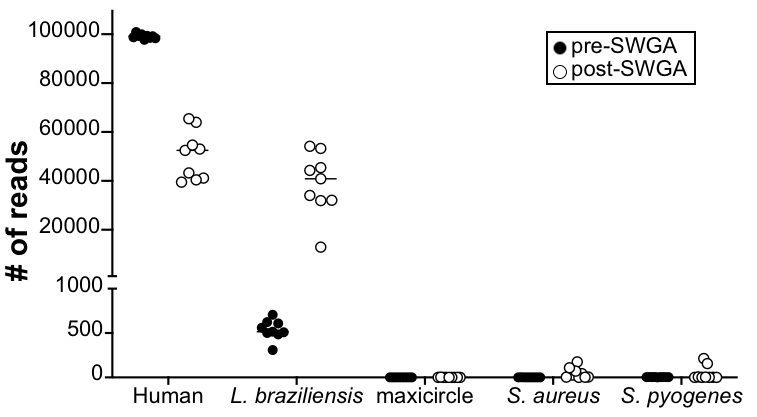

Supplement: S1 Fig — Plot showing number of reads (out of 100,000 subsampled reads) from each of 9 patient samples (points) that mapped to genomes of human, L. braziliensis, Staphylococcus aureus, Streptococcus pyogenes, and the L. braziliensis kinetoplast maxicircle. (TIFF) [file ppat.1011230.s001.tiff]

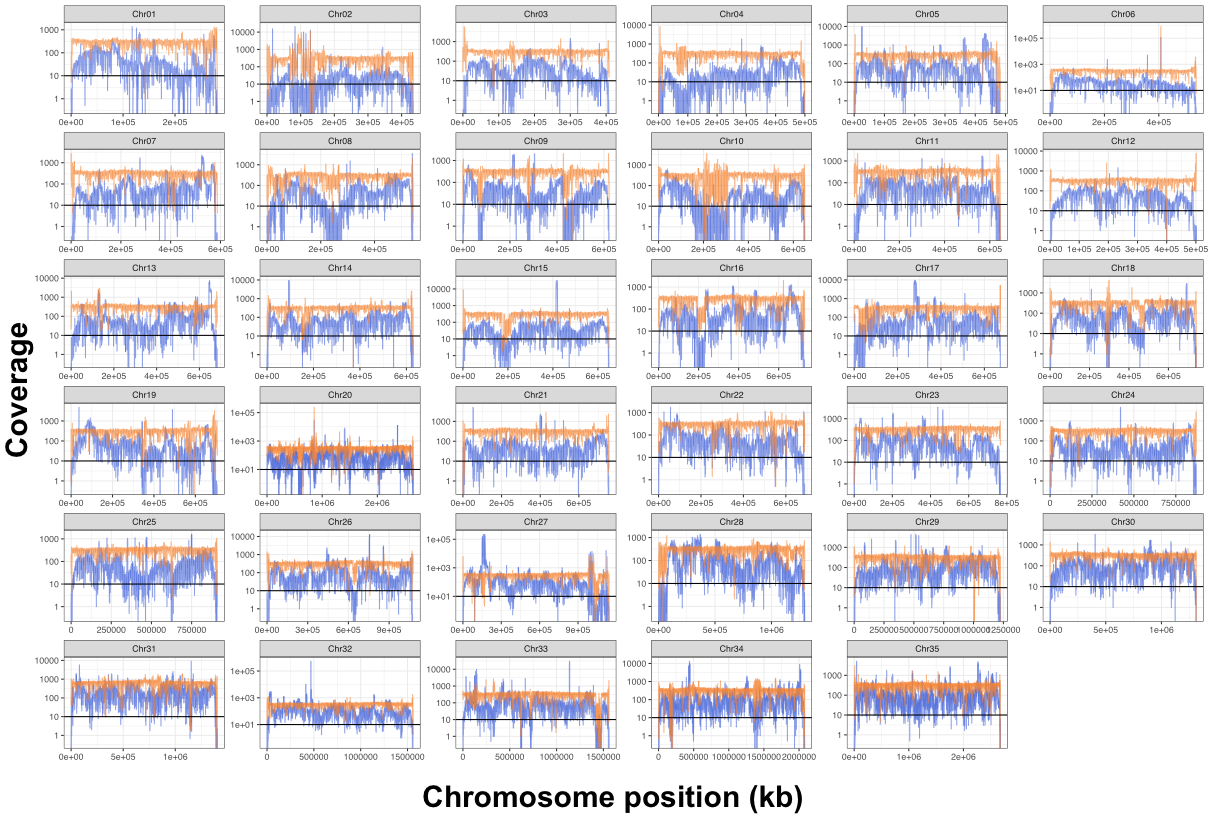

Supplement: S2 Fig — Coverage plots for 35 L. braziliensis chromosomes in SWGA data from a single patient (#7; blue lines) compared to whole genome sequencing (WGS) of pure, cultured L. braziliensis (orange lines). Data were merged from all SWGA primer sets to maximize coverage. (TIFF) [file ppat.1011230.s002.tiff]
