# Supplementary material for: Selective whole-genome amplification reveals population genetics of Leishmania braziliensis directly from patient skin biopsies
Source: PLoS Pathog. 2023 Mar 20;19(3):e1011230. doi: 10.1371/journal.ppat.1011230 (PMC10063166; doi:10.1371/journal.ppat.1011230)
Supplement: Attachment — Submitted filename: SWGA_Microbiome_rebuttal.docx [file ppat.1011230.s007.docx]

Dear Dr. Wetzel, Dr. Phillips, Dr. Haldar, and Dr. Malim,

Attached please find the revised manuscript entitled “**Selective whole-genome amplification reveals population genetics of *Leishmania braziliensis* directly from patient skin biopsies**”. Thank you for the opportunity to respond to the reviewer’s comments. We were pleased that they viewed our study as “well written” (both reviewers), “a very interesting, useful and feasible method” (reviewer 1), and “the results are well controlled and clearly presented” and that we “make a convincing case that SWGA will be useful for the Leishmania community” (reviewer 2). Below, we provide a point-by-point rebuttal addressing each of the reviewer’s comments. Our revisions to the manuscript include reframing the second half of the paper as a proof-of-concept, a revised figure 3 that now shows the utility of SWGA for somy analysis, and a new supplemental figure that validates SWGA primer design, along with clearer language throughout. Changes to the manuscript are tracked, and we have included a ‘clean’ copy of the revised manuscript for easier reading. We thank both reviewers for their time and their constructive feedback – their input has undoubtedly improved the manuscript.

**Editor’s Remarks:**

**Q1. “***In particular, as the reviewers indicate, the impact of this work would be significantly increased by analyzing any correlations between genetic profiles provided and treatment outcome. This should be done by 1) comparing/contrasting sequencing results documented in the manuscript for the strains/biopsies linked to treatment success or failure to existing sequencing data from available genomes (from more susceptible or more resistant strains) and 2) validating susceptibility results in vitro for a subset of identified clinically resistant or susceptible variants. If sample size was not sufficiently powered to draw strong conclusions about relationships between variants and outcome/resistance (as one reviewer suggests), this section could be presented as a proof-of-concept exercise to demonstrate the feasibility of applying SWGA to studies associating clinical phenotypes and parasite genetics.”*

**A1.** Unfortunately, no information on treatment outcome was made available for the public *L. braziliensis* WGS data. We apologize that this was not clear in the paper. As a result, the only treatment outcome data available is from the 18 new SWGA samples we reported in the paper. With this small sample size, it is very unlikely that quantitative genetics approaches would yield significant insights. For example, our previous analysis to associate clinical outcome with parasite diversity with *L. infantum* with 109 samples showed low power from GWAS and wide confidence intervals for heritability [(1)](https://sciwheel.com/work/citation?ids=14295434&pre=&suf=&sa=0&dbf=0), so 18 samples is certainly not sufficient. Thus, the latter part of the manuscript has been rewritten as a proof-of-concept for how this method can be used to readily identify SNPs associated with a covariate of interest.

**Reviewer #1**:

**Q1.** *“It is very difficult to follow the description of the results section on SNP analyses in the context of therapeutic failures. The mix of samples and the lack of clarity of exclusion of some of them makes it hard to define the extent to which conclusions in relation to outcome could be drawn. As a first step I would request a flow chart of samples, and the process of inclusion/exclusion for analysis regarding outcome. Then the clinical and demographic metadata should be clearly defined from isolates as well as biopsies, so that the reader can contrast the quality of the definitions of outcome and therefore the interpretability of the data.”*

**A1.** We apologize for the confusion. Unfortunately, the 41 publicly available WGS genomes from across S. America used in our population genomic analyses **(Figure 5)** do not have treatment outcomes reported in their respective studies. Based on recommendations from the reviewers, we have shifted the emphasis of our results to focus on a proof-of-concept exercise where we show that SWGA could be used to identify SNPs associated with specific geographic regions. We have significantly edited the results and discussion to reflect this change.

**Q2.** *“If any relationships are to be drawn in relation to susceptibility, at least some of the variants should be validated at least in vitro, since the authors are talking about “parasite drug resistance.* *I strongly suggest that the scope of the paper be the method and the potential applicability, more than the relationship of variants with outcome and/or resistance. The latter seems to stem from a secondary analysis of the data that is not really powered to draw the expected conclusions, and could be better presented as an “academic” exercise of feasibility of implementing this method to association studies of clinical phenotypes and parasite genetics.”*

**A2.** We agree that we are significantly underpowered to draw firm conclusions about SNPs linked to clinical outcome (we expect sample sizes closer to 100 would be required for this, as we describe above), and it was our intent with the initial submission for this to be viewed as a proof-of-concept, and we apologize that this was not made more clear in the initial submission. To reinforce this notion, we have rewritten the relevant sections of the results and discussion to shift our focus from the relationship of variants with treatment outcome and/or resistance to a focus on the method and potential application.

**Q3.** *“It would be interesting to show coverage of the selectively amplified DNA for bacterial commensals to show that this amplification tool is really specific to Leishmania DNA and that the use of S. aureus genome as background was indeed useful and successful.”*

**A3.** There are two situations in which a genome should not be amplified in SWGA. 1) a genome is included in the background during SWGA primer design (as was the case for *S. aureus* and *S. pyogenes*); or 2) a genome is excluded altogether from the primer design (as was the case for the kinetoplast genome). We have now included an analysis of pre- and post-SWGA from patient samples for *S. aureus*, *S. pyogenes* and the kinetoplast genome to formally evaluate whether one or both methods resulted in true selectively of our SWGA primers. We now show in a new supplementary figure **(Supplementary Figure 1)** that our primers are highly selective for *L. braziliensis* over genomes included in the background or excluded from the analysis entirely. These data are now described in the second paragraph of the results **(page 5, lines 8-16)**, and we have updated the methods to explain how this analysis was done **(page 10, lines 43-46)**.

**Q4.** *“Not clear why the somy analyses from clinical samples were not as conclusive as isolated parasites. How can high host DNA content affect somy analyses?”*

**A4.** It is not the high host DNA content directly, but the stochastic coverage of SGWA that affects somy analysis. The simplest method to estimate somy is via read coverage (supernumerary chromosomes will have more coverage). Unfortunately, SWGA produces more stochastic read coverage than WGS both *within* a chromosome **(see Figure 2)**, and between chromosomes of the same sample, including a bias away from small chromosomes. Such biases render the coverage method less reliable. The other common approach is to look for heterozygous SNP sites that deviate from the expected 1:1 ratio of reads that ‘call’ each of the two alleles (eg: in a AT heterozygote site with 60x coverage we expect 30A: 30T in a diploid chromosome). Supernumerary chromosomes will deviate from this (for example, a trisomic chromosome may have a 2:1 ratio such as 40A: 20T). Stochastic read coverage also affects this approach **(Figure 3)**. Since our initial submission we have revisited this analysis and found that by increasing the stringency of our SNP filtering, we were able to significantly improve somy analysis for SWGA. Specifically, we increased the required reads/allele from ≥ 5, to ≥ 10. This means that, to be considered in the somy analysis, a SNP must have at least 10 reads matching the reference and at least 10 reads for an allele that differs from the reference. In addition to this more stringent analysis, we also included a filter that requires an overall read depth at each SNP of greater than 30 and less than 200. As a result of these changes, we have completely revised **Figure 3**, which now shows that somy analysis of patient samples data is possible with SWGA, and we provide evidence of non-diploid population states in multiple patient samples. The data is complex, because of the uneven coverage of SWGA and because somy analysis performed in this way relies upon sufficient heterozygous SNPs. Managing this complexity is possible, but we think it is beyond the scope of this proof-of-principle analysis. We have updated the methods with these improved filtering parameters **(page 12, lines 11-12).**

**Q5.** *“Figure 4 is unnecessary”*

**A5.** We have combined Figure 4 with Table 1, which together form a new **Figure 4**. This creates a more cohesive figure that illustrates the workflow for screening large numbers of samples by SWGA, alongside the metrics for the genomes that resulted from this screen. We believe this figure in necessary to explain our workflow for using SWGA at the scale of hundreds of samples.

**Q6.** *“I believe the argument for lack of L. braziliensis sequences (discussion section) is not lack of infrastructure or difficulty in isolation, but rather a limitation of technical capacity and the most important, lack of economic resources for expensive sequencing projects.”*

**A6.** Yes, we agree that there are likely multiple factors that contribute to a limited number of *L. braziliensis* genomes. We included as statement pointing to disparity in technical capacity and lack of resources for sequencing **(page 3, line23; and page 8, lines 29-30).**

**Reviewer #2**

**Q1.** *“Discussion: While the advantages of SWGA over the more cost-intensive, commercially available solutions are convincingly presented throughout the manuscript, a more critical discussion of the limitations of SWGA compared to Sure Select arrays should be added. This would also allow to better highlight the strengths and applications of SWGA. How are the methods comparing with respect to technical background and quantitative genome assessment on for example gene and chromosome CNVs, or variations in SNP frequencies?”*

**A1.** Good point. We have now expanded the first paragraph of our discussion to include a more thorough description of the limitations of SWGA **(page 8, lines 33-39)**, resulting in a more balanced view of the pros and cons of SWGA and SureSelect. For example, we now highlight that the Sure Select method is likely more sensitive than SWGA and can amplify parasite genomes from samples with as low as 0.006% *Leishmania* DNA [(2)](https://sciwheel.com/work/citation?ids=7964790&pre=&suf=&sa=0&dbf=0). This could be important for clinical samples from patients with mucosal leishmaniasis, or those that cure disease after one round of antimony treatment, since both groups of patients have been shown to have very low parasitemia. We also highlight that, in theory, the performance of SureSelect technology should be independent of host background, whereas SWGA primers may need to be redesigned for host backgrounds that differ dramatically from human, such as the sandfly vector.

**Q2.** *“Figure 2: Panel 2B should be either discussed at the beginning (it is discussed at the very end of this chapter) or the panel labeling and organization should be adapted and come at last. The data shown in panel 2D should be more critically assessed and the cause (technical) and consequences (no quantitative genetic information possible) of the important coverage fluctuations seen across the entire chromosomes outside the ‘complicated’ areas should be discussed.”*

**A2**. We have moved the description of the mouse SWGA results (originally Figure 2B) to the beginning of the ‘Validation of SWGA assay on mouse and primary human samples’ section (**now Figure 2A**). We have also included a statement that points to SWGA as the cause of uneven coverage across the chromosomes due to its multiple displacement amplification (MDA) and that this limits our ability to perform quantitative genetic tests **(page 5, lines 30-32)**.

**Q3**. *“Figure 3: There is a misconception here: a SNP frequency ratio of 0.5 is not proof of a disomic state! Here, populations are sequenced and thus the somie values of each individual parasite are integrated in the final genome sequence. Thus, if the trisomy is caused by either of the two haplotypes, the heterozygous SNPs will be balanced again at 0.5 in the population. Also, for a meaningful analysis of SNP frequencies, a robust assessment of the read depth needs to be available, which is not the case for SWGA. I would remove this figure or add it to supp data, indicating that SWGA is less useful in estimating somie differences based on either read depth variations or SNP frequency shifts.”*

**A3.** We agree that mosaic aneuploidy within infections (or cultures) can cause differential fluctuations in the numbers of haplotype copies in different cells, which could in theory lead to within sample allele counts similar to 0.5 when there are only two haplotypes. Similarly, disomic chromosome SNP frequency could in theory differ from 0.5 if haplotype selection [(3)](https://sciwheel.com/work/citation?ids=4752709&pre=&suf=&sa=0&dbf=0) is occurring, reducing the cells that contain one of the two haplotypes. However, based on our experience with Trypanosomatids, there is usually a strong concordance between the Read Depth and Allele frequency analysis. The most parsimonious explanation for observing a consistent pattern across many SNPs within a chromosome that falls to a within-sample SNP frequency of 0.5 is a disomic state. This is because most SNPs within natural population are rare, so many are unique to a single clone. Thus when complex multi-clonal populations are sequenced, we would expect to observe either ratios of 0.25, 0.5 and 0.75 (for simple case with two clones or tetrasomy) or an even wider, more complex distribution for higher clone number of higher somy (see figure 1 from chapter 5 of [(4)](https://sciwheel.com/work/citation?ids=14275200&pre=&suf=&sa=0&dbf=0)). Somy can also be estimated via coverage, and our expectations of somy are consistent between these two aspects of the data [(5)](https://sciwheel.com/work/citation?ids=12975565&pre=&suf=&sa=0&dbf=0). For these reasons, we believe that our somy analysis should remain as a main figure in the manuscript. We have edited the text to highlight the limitations of only using the SNP frequency to estimate somy **(Page 5, Lines 44-45)**. Regarding the read depth, we have improved our SNP-position filtering for the somy analysis, now only using positions where both of alleles had at least 10 reads, and the total read depth in the position was at least 30 and lower than 200. This improved the shape of the allele read depth distributions, as seen in the updated **figure 3**.

**Q4.** *“Figure 4: How does the nested procedure – i.e. a first shallow sequencing followed by sequencing of pooled libraries – affect SNP frequencies and read depth (see comments above)? Shouldn’t this introduce a major bias given that the initially obtained 20% includes both unique (hence the pooling), but also overlapping regions that will then be over-represented in the second round of sequencing? How does this affect the more quantitative aspects of comparative genome analyses, which are at the core of biomarker discovery? These drawbacks need to be discussed.”*

**A4.** We think there may be some confusion here as to what happens between shallow and deep sequencing. First, each SWGA reaction is used to prepare a separate library for sequencing. All libraries are barcoded and pooled (multiplexed) for a shallow sequencing run, which serves only to identify those reactions with ≥ 20% parasite mapping reads. In other words, the shallowing sequencing run allows us to quickly screen many SWGA reactions (hundreds) to determine which ones ‘worked’. Once these successful reactions are identified, we do not resequence the multiplexed pool. Instead, we return to the original libraries and create a new multiplexed pool containing only those reactions that were deemed successful, which are then subjected to deep sequencing. This is not nesting, and the original shallow sequencing has no impact on the deep sequencing one, other than to inform us which samples might be best to deep sequence. Second, since we cannot predict which primer set will work best for each patient sample, we subject each patient sample to SWGA with multiple primer sets. Sometimes, this leads to a situation where a single patient sample has multiple successful SWGA reactions. In this case, these reactions would be sequenced separately, and the resulting sequencing data would be concatenated. This is not nesting either and will not influence downstream comparative genomics.

**Q5.** *“Figure 5 panel E: Define the triangle and the black dot in the legend. Based on the color code, all SWGA samples are clustering together. This is not properly highlighted in the results section. This signal should be pointed out and a role (even if minor) of SWGA on clustering should be discussed.”*

**A5.** We apologize for this omission. We have now included the triangle and black dot in the figure key and legend **(Figure 5E)**. Even though our SWGA genomes cluster together (which we now clearly state in the results **(page 7, lines 17-23)** we do not believe this is due to technical reasons associated with the SWGA method. Our comparisons of the same cultured *L. braziliensis* isolate analyzed by SWGA and WGS show no separation on the PCA or tree, despite technical differences between SWGA and WGS. Furthermore, our SWGA genomes form a monophyletic clade that includes 4 WGS genomes – the only genomes in our analysis from the same state in Brazil as our SWGA genomes. These data suggest that geography, rather than method, is the primary driver of the clustering observed in Figure 5. However, it is still reasonable to question whether the uneven coverage we observed in SWGA **(Figure 2D)** has any meaningful impact on clustering. To formally test this, we simulated the loss of signal (SNPs) across genome and evaluated whether the structure of the tree changed. The *L. braziliensis* reference genome was split into 10 kb segments and we discarded SNP data from 20%, 40%, and 60% of these 10kb segments and regenerated the tree **(Supplementary Figure 3)**. These data show that even after 20% and 40% signal loss, our 18 SWGA samples still form a monophyletic clade with the 4 other Bahia samples (WGS) [(6)](https://sciwheel.com/work/citation?ids=10572604&pre=&suf=&sa=0&dbf=0). After dropping 60% of the SNPs we finally see the Bahia WGS and our Bahia SWGA samples split off into sister clades. We expect that the robustness of our clustering – even under these simulations of extreme signal loss – is due to high genetic diversity in *L. braziliensis* and thus the large number of SNPs (nearly 900,000; see figure 5 legend) used to construct this tree.

**Q6.** *“Figure 6: The functional enrichment observed for the structural mutants that change the protein sequence changes is potentially very interesting! The fact that half of the unique variants are contributed by the SWGA samples is a bit concerning – how sure is it that these are not introduced by the method?”*

**A6.** SWGA is not known to introduce SNPs, inversions or deletions during multiple displacement amplification [(7,8)](https://sciwheel.com/work/citation?ids=13531081,13531085&pre=&pre=&suf=&suf=&sa=0,0&dbf=0&dbf=0). For example, genomes of *P. vivax* had SNP rates, transition-to-transversion ratios, and nonsynonymous-to-synonymous ratios that were nearly identical between SWGA and WGS [(9)](https://sciwheel.com/work/citation?ids=3932759&pre=&suf=&sa=0&dbf=0). Similarly, *P. falciparum* genomes generated from matched venous blood (VB) by WGS and dried blood spots (DBS) by SWGA showed “very high concordance in population genetic data between VB and DBS samples” [(10)](https://sciwheel.com/work/citation?ids=3051667&pre=&suf=&sa=0&dbf=0). To formally test whether our SWGA samples were enriched for indels, we examined the indel to SNP ratio for 4 SWGA genomes from Bahia, 4 WGS genomes from Bahia, 4 WGS genomes from Pernambuco, and 4 WGS genomes from Peru. We found the INDEL to SNP ratio to be 0.256, 0.205, 0.217, and 0.179, respectively. Similarly, when we carried out WGS and SWGA on the same sample of pure cultured parasites, we obtained INDEL to SNP ratios of 0.251 and 0.219, respectively. Taken together, these data suggest a generally higher trend for this ratio in genomes from Northeast Brazil, rather than a technical issue that results in more indels in our SWGA genomes. Finally, only a single panel of our results focuses on INDELS **(Figure 6B)** and the remaining results use only SNPs, which is common practice for many population genetic studies. We have now included these data in the results section **(page 7, lines 41-46).**

**Q7.** *It is unclear to me how many samples show mutations in a given gene (same position or different), which would indicate convergence and thus selection. Have any of these genes been previously linked to treatment failure? I am surprised that these changes were not correlated to the observed and documented outcome of treatment across the samples with available genomes (including the 59 genomes sequenced by others). Is there any correlation, even if statistically insignificant?”*

**A7.** Unfortunately, no treatment outcome data was reported for any of the patients from which the 41 genomes were previously published. In addition, of our 18 SWGA genomes (for which we do have treatment outcome data), only 3 were from patients that cured after a single round of therapy and the remaining 15 are from patients that failed the initial round of therapy. Thus, we are underpowered to find variants linked to treatment outcome. In the discussion, we suggest a possible reason why it may be particularly challenging to SWGA parasite genomes from patients who ultimately respond well to chemotherapy **(page 9, beginning line 16)**. We have reworked our results and discussion to make it clear that the final two figures of the paper are proof-of-concept, and we have shifted the emphasis from finding SNPs linked to treatment outcome, to finding SNPs that are enriched in isolates from the Northeast region of Brazil.

**Bibliography**

[1.    Grace CA, Sousa Carvalho KS, Sousa Lima MI, Costa Silva V, Reis-Cunha JL, Brune MJ, et al. Parasite Genotype Is a Major Predictor of Mortality from Visceral Leishmaniasis. MBio. 2022 Oct 12;13(6):e0206822.](https://sciwheel.com/work/bibliography/14295434)

[2.    Domagalska MA, Imamura H, Sanders M, Van den Broeck F, Bhattarai NR, Vanaerschot M, et al. Genomes of Leishmania parasites directly sequenced from patients with visceral leishmaniasis in the Indian subcontinent. PLoS Negl Trop Dis. 2019 Dec 12;13(12):e0007900.](https://sciwheel.com/work/bibliography/7964790)

[3.    Prieto Barja P, Pescher P, Bussotti G, Dumetz F, Imamura H, Kedra D, et al. Haplotype selection as an adaptive mechanism in the protozoan pathogen Leishmania donovani. Nat Ecol Evol. 2017 Dec;1(12):1961–9.](https://sciwheel.com/work/bibliography/4752709)

[4.    Reis-Cunha JL, Bartholomeu DC. Trypanosoma cruzi Genome Assemblies: Challenges and Milestones of Assembling a Highly Repetitive and Complex Genome. Methods Mol Biol. 2019;1955:1–22.](https://sciwheel.com/work/bibliography/14275200)

[5.    Briggs EM, Marques CA, Reis-Cunha J, Black J, Campbell S, Damasceno J, et al. Next-Generation Analysis of Trypanosomatid Genome Stability and Instability. Methods Mol Biol. 2020;2116:225–62.](https://sciwheel.com/work/bibliography/12975565)

[6.    Alves-Ferreira EVC, Toledo JS, De Oliveira AHC, Ferreira TR, Ruy PC, Pinzan CF, et al. Differential Gene Expression and Infection Profiles of Cutaneous and Mucosal Leishmania braziliensis Isolates from the Same Patient. PLoS Negl Trop Dis. 2015 Sep 14;9(9):e0004018.](https://sciwheel.com/work/bibliography/10572604)

[7.    Cocking JH, Deberg M, Schupp J, Sahl J, Wiggins K, Porty A, et al. Selective whole genome amplification and sequencing of Coxiella burnetii directly from environmental samples. Genomics. 2020 Mar;112(2):1872–8.](https://sciwheel.com/work/bibliography/13531081)

[8.    Thurlow CM, Joseph SJ, Ganova-Raeva L, Katz SS, Pereira L, Chen C, et al. Selective Whole-Genome Amplification as a Tool to Enrich Specimens with Low Treponema pallidum Genomic DNA Copies for Whole-Genome Sequencing. mSphere. 2022 Jun 29;7(3):e0000922.](https://sciwheel.com/work/bibliography/13531085)

[9.    Cowell AN, Loy DE, Sundararaman SA, Valdivia H, Fisch K, Lescano AG, et al. Selective Whole-Genome Amplification Is a Robust Method That Enables Scalable Whole-Genome Sequencing of Plasmodium vivax from Unprocessed Clinical Samples. MBio. 2017 Feb 7;8(1).](https://sciwheel.com/work/bibliography/3932759)

[10.   Oyola SO, Ariani CV, Hamilton WL, Kekre M, Amenga-Etego LN, Ghansah A, et al. Whole genome sequencing of Plasmodium falciparum from dried blood spots using selective whole genome amplification. Malar J. 2016 Dec 20;15(1):597.](https://sciwheel.com/work/bibliography/3051667)
